# Reactivation of Epstein–Barr Virus Presenting as Massive Splenomegaly after Initiation of Golimumab Treatment

**DOI:** 10.1155/2020/3641813

**Published:** 2020-04-10

**Authors:** Anthony J. Febres-Aldana, Christopher A. Febres-Aldana, Kathrin Dvir, Gliceida Galarza-Fortuna, Michael Schwartz, Ana M. Medina, Vathany Sriganeshan

**Affiliations:** ^1^Department of Internal Medicine, Mount Sinai Medical Center, Miami Beach, FL 33140, USA; ^2^Arkadi M. Rywlin Department of Pathology and Laboratory Medicine, Mount Sinai Medical Center, Miami Beach, FL 33140, USA; ^3^Division of Oncology, Department of Internal Medicine, Mount Sinai Medical Center, Miami Beach, FL 33140, USA; ^4^Herbert Wertheim College of Medicine, Florida International University, Miami 33199, FL, USA

## Abstract

Epstein–Barr virus infection is most commonly asymptomatic in the acute setting, where the end result of infection is the adoption of a viral latency phenotype. The virus can reactivate later in life leading to the abnormal proliferation of the infected B, T, or NK cells. Hereby, we report a 71-year-old female with seronegative rheumatoid arthritis who presented with massive splenomegaly, pancytopenia, and positivization of antibodies against double-stranded deoxyribonucleic acid (dsDNA) after initiation of the anti-tumor necrosis factor (TNF) golimumab. The diagnosis of EBV-associated lymphoproliferative disorder (LPD) was demonstrated by elevation of the plasmatic EBV viral load. Withdrawal of the anti-TNF and treatment with the anti-CD20 antibody rituximab were able to revert the clinical abnormalities. EBV-associated LPDs are described after initiation of other anti-TNF agents, such as infliximab, but no reports of golimumab-associated EBV LPD are found in the literature. The mechanisms for this occurrence are not clear, but these are known to involve expression of a panel of viral proteins specific to the viral latency phenotypes.

## 1. Introduction

Epstein–Barr virus (EBV) is a gamma-herpesvirus that prevails in over 90% of the population. The primary infection is most commonly asymptomatic, and it may manifest later in adulthood [1]. Although B cells are the main target of EBV due to its tropism for CD21^+^ cells, the virus can also infect T cells, NK cells, and less frequently epithelial cells. The virus may remain dormant in these cells and may reactivate later in adulthood through mechanisms that are poorly understood. This article reports the occurrence of EBV reactivation presenting as a biclonal lymphoproliferative disorder (LPD) in a patient with rheumatoid arthritis, triggered by initiating therapy with the anti-tumor necrosis factor (TNF) golimumab.

## 2. Case Presentation

A 71-year-old woman presented to our emergency department because of left-sided abdominal pain, fatigue, anorexia, early satiety, and low-grade fever for two weeks. She carried the diagnosis of seronegative rheumatoid arthritis (RA) based on the presence of inflammatory arthritis with negative anticitrullinated peptides antibodies (ACPA) and negative rheumatoid factors (RF). Her inflammatory symptoms were initially controlled on etanercept, but the medication was switched to tofacitinib a year prior to presentation due to chronic cough. However, tofacitinib triggered episodes of elevated blood pressure, dizziness, and headaches, so golimumab was started instead three months before. While on golimumab, her symptoms related to the arthritis were controlled. Her other medications included metoprolol tartrate, amlodipine, irbesartan, levothyroxine, and acetaminophen for arthralgias. She had recently come back from South Africa where she visited only urban areas. Her family history was remarkable for a sister with inflammatory bowel disease and essential thrombocythemia. In contrast to her sister, the patient never presented symptoms consistent with inflammatory bowel disease or psoriasis.

**Figure 1:**
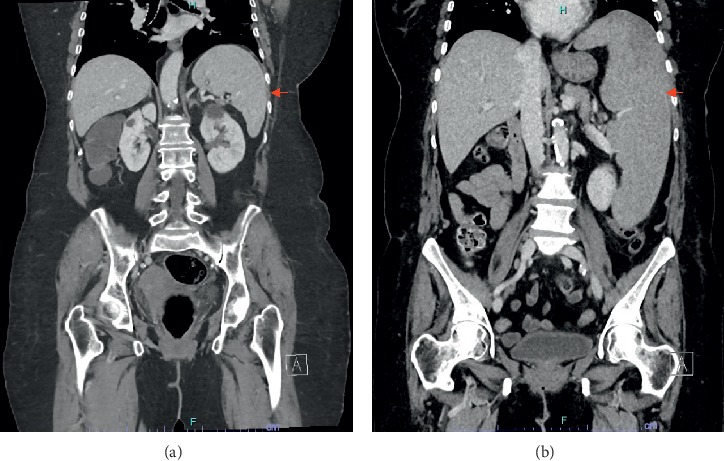
Coronal views of computer tomography scan of the abdomen performed one month before (a) and three months after (b) starting therapy with golimumab demonstrating the massive enlargement of the spleen (arrow).

**Figure 2:**
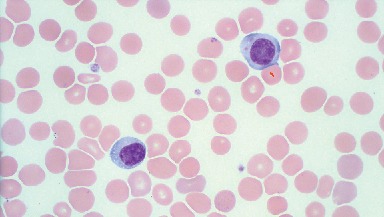
Peripheral blood smear showing atypical lymphocytes with abundant cytoplasm, hugging of a red blood cell (arrow), tiny cytoplasmic vacuoles, and mature chromatin, resembling Downey type II cells seen in acute infectious mononucleosis. Staining: Wright-Giemsa; magnification: 100x.

On presentation, her vital signs were within normal limit, and examination revealed edema of lower extremities and a palpable spleen. Laboratory tests were remarkable for a hemoglobin of 8.0 g/dL with a normal mean corpuscular volume and an increased percentage of reticulocytes at 5.27% with a negative direct antiglobulin test. Platelet count was 4.4 × 10^10^/L, and white blood cell count was 6.49 × 10^9^/L with 27% of atypical lymphocytes. These parameters were normal before starting golimumab. Serum chemistry was normal except for a mild elevation of alkaline phosphatase of 178 IU/L (range of reference 45–117 IU/L) and a lactate dehydrogenase of 641 IU/L (range of reference: 84–246 IU/L). Iron studies revealed normal iron, transferrin, and ferritin, and haptoglobin was undetectable. Her C-reactive protein was elevated at 99.1 mg/L. Anti-double-stranded deoxyribonucleic acid (DNA) antibody determined by the *Crithidia luciliae* indirect immunofluorescence assay was positive at 1 : 20. Other antinuclear antibodies were negative. The patient was admitted to the medical ward. An abdominal computed tomography (CT) scan demonstrated the presence of massive splenomegaly (Figure 1), with focal hypoattenuation and normal uptake on positron emission tomography (PET) scan. The levels of C3 were 70 mg/L, and C4 levels were within normal limits. Peripheral blood smear revealed the presence of Downey type II cells (Figure 2), and an interferon-*γ* release assay was negative. A bone marrow biopsy revealed a hypercellular bone marrow for age with trilineage hematopoiesis, erythroid hyperplasia, and mild reticulin fibrosis. Flow cytometry of the blood showed that the lymphocytosis was composed mainly of CD4^+^ T-lymphocytes with no aberrancy and 10% of B cells. The presence of reactive lymphocytes prompted testing for viral infections. EBV viral capsid antigen (VCA) immunoglobulin (Ig) G was 207 IU/mL, and EBV-determined nuclear antigen (EBNA) IgG was 71.1 IU/mL, with a negative EBV VCA IgM and a positive CMV IgG with a negative CMV IgM. A quantitative PCR of EBV DNA on peripheral blood mononuclear cells was positive at 1,703 copies/mL. No prior EBV viral load was performed before presentation. The patient was transfused one unit of red blood cells, golimumab was stopped, and she was discharged home.

**Figure 3:**
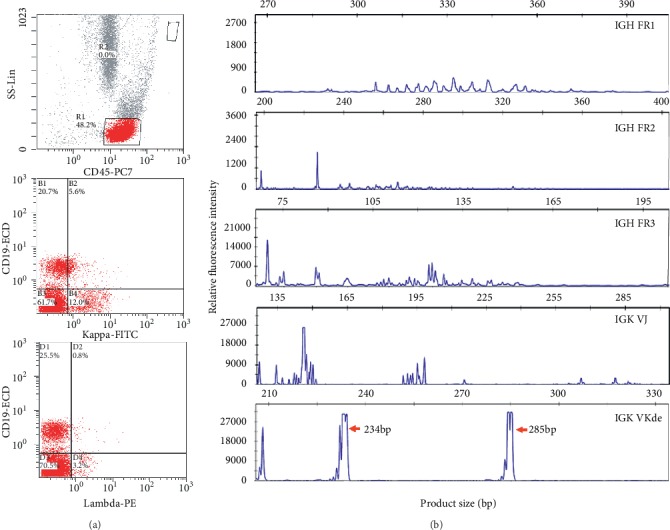
Flow cytometry (a) and B-cell immunoglobulin gene rearrangement studies (b) demonstrating the clonal nature of the golimumab-induced lymphoproliferative disorder. An abnormal population of B cells (CD-45+, CD-19+) with a dim expression of surface kappa light chain and negative for lambda light chain. B electropherograms of fluorescently labeled PCR products detected by capillary gel electrophoresis showing polyclonality, Gaussian distribution of products, using the BIOMED-2 primer sets *IGH* FR 1, FR2, FR3, *IGK VJ*, and *IGK VKde*. Two broad dominant peaks (234.06 bp and 285.08 bp) were identified with the *IGK VKde* primer set, which targets the deleting element of the kappa light chain. This result is consistent with the presence of two B-cell clones.

Six weeks later, she presented to the office with partial improvement in her initial symptoms and decrease in her spleen size. Repeat flow cytometry revealed that the B cells doubled to 20–25% (Figure 3(a)), biclonal for the *IGK* gene (Figure 3(b)) on B-cell receptor (BCR) gene rearrangement studies. The diagnosis showed iatrogenic EBV-associated B-cell lymphoproliferative disorder (LPD), and rituximab was started. After two months of treatment, blood cell counts normalized and the spleen became nonpalpable. On subsequent follow-ups, flow cytometry of the peripheral blood showed no clonal population of B cells, and EBV viral load remained negative. Anti-dsDNA persisted positive with a 1 : 80 dilution.

## 3. Discussion

The patient developed an immunodeficiency-associated (IDA) LPD triggered by reactivation of EBV after initiation of the anti-tumor necrosis factor (TNF) golimumab. LPD is an umbrella term that ranges from reactive to monoclonal lymphoid proliferations with malignant behavior [2], and this reflects the stage of the EBV replication cycle [3]. Even though the precise events that lead to the expression of the viral latency phenotypes are not well defined, these are identified based on the expression of multiple nuclear antigens (EBNA) and latent membrane proteins (LMP), proteins that help the virus to evade immune surveillance. The combination of different isoforms of each of these proteins and transcripts allows the distinction of at least three well-defined latency patterns with specific clinical expressions [3, 4]. The patient's LPD presentation was consistent with expression of a latency III phenotype, which is associated with IDA-LPDs, and it is the most immunogenic result of expression of all EBV latency transcripts and proteins [3]. The restricted expression of EBV-encoded small RNAs (EBER) or EBNA1 corresponds to pattern I latency, and it occurs in memory B cells. Selected expression of EBNA1, LMP1, LMP2A and LMP2B, EBERs, and the BamHI A rightward transcripts (BARTs) defines pattern II latency. This pattern is seen in germinal center B cells. Latency III pattern occurs in naïve B cells [3].

Exclusive latency I phenotype occurs in subclinical reactive lymphoid hyperplasia, Burkitt lymphoma, plasmablastic lymphoma, and primary effusion lymphoma. Coinfection of EBV in latency I pattern with human herpesvirus type 8 and human immunodeficiency virus (HIV) can lead to the development of a monoclonal LPD. Latency II pattern occurs in otherwise immunocompetent individuals who develop EBV-associated Hodgkin lymphoma, T/NK-cell lymphomas, and follicular T-cell lymphoma [3]. This type of latency also occurs during the hydroa vacciniforme and severe mosquito bite allergy, both polyclonal LPDs localized to the skin and with a potential to disseminate as a clone-restricted LPD [5]. Latency III occurs in the case of EBV-associated diffuse large B-cell lymphoma, posttransplant lymphoproliferative disorders, immunodeficiency-associated LPDs, and iatrogenic LPDs. Expression of the latency III phenotype is associated with simultaneous inhibition of apoptosis and progression of the cell cycle to M phase, with the result of growth arrest and consequent immortalization of the infected lymphocytes [5].

EBV reactivation can be linked to the use of TNF blockers [6], with infliximab being the most common culprit agent in the class [7, 8]. However, the evidence linking the use of TNF agents as an independent risk factor for the development of LPDs is nonconclusive, with some cohorts demonstrating an increased risk for development of lymphomas [9] but others demonstrating a nonsignificant association [10, 11]. This discrepancy could be explained by the degree of disease activity of the RA, including history of previously uncontrolled disease before starting the TNF blocker, as RA is associated independently with the development of LPDs, including lymphomas, and the risk seems not to be fully reversed after the use of any of the disease modifying antirheumatic drugs (DMARDs) [12]. In fact, these patients have higher risk for lymphoma development even when on treatment [13]. The disease of our patient presented a low activity based on her symptoms related to the RA at the moment of initiation of golimumab, and the decision to start the biologic agent was driven by the fact that she had poor response to other DAMRDs in the past, including methotrexate (MTX). In regards to those cases that occur in association with reactivation of EBV, this seems to be a rare event and therefore the association is more difficult to determine. This is the first report of a case of an EBV-associated LPD after starting golimumab in a patient who was previously exposed in two separate moments to a Janus Kinase inhibitor and to etanercept and that had clinical reversion after its withdrawal. The regression of the LPD after withdrawal of therapy has also been described for cases of RA-associated EBV-lymphomas in patients on MTX [14], with the presence of EBV in the tumor being a predictor of the occurrence of this phenomenon. It is unlikely that tofacitinib had played a role in this case as the agent has not proven to alter EBV or CMV viral loads [15]. Of note, no studies evaluating the effect of tofacitinib on EBV viral load in patients with RA have been performed to date and the data available come from patients with psoriasis, disease which has not intrinsic association with the development of lymphomas. One potential contributing factor to this clinical presentation was the history of long-standing use of etanercept by the patient, which stopped months before initiating golimumab, as the long-term side effects of use of these agents are not predictable [16].

For the diagnosis, the EBV load in peripheral blood mononuclear cells (PBMC) can aid to determine when the LPD is driven by EBV reactivation. While an EBV viral load above 500 copies per 500 ng determines an association of EBV reactivation with the LPD [17], the patients with RA tend to have viral loads that are around 10-fold higher than those patients without the disease [18]. The use of anti-TNF therapy by itself is not associated with an increased EBV load [19, 20]. Therefore, the kinetics of the EBV viral load has a better predictive value for the diagnosis of EBV-associated LPDs when the PBMC EBV viral load is monitored, namely in patients who are allograft or hematopoietic stem cell transplant (HSCT) recipients [21]. B-cell receptor (BCR) rearrangement studies are useful in these cases as well as they allow the identification of clonal nature of the LPD, as most EBV-related LPDs are B cells in origin [2]. Except for the reactive lymphoid proliferations, EBV-associated LPDs are most commonly monoclonal. Oligoclonal or polyclonal LPDs are less commonly seen.

Expression of viral proteins during EBV reactivation may result in the cross-activation of self-reactive T or B cells, a phenomenon termed antigenic mimicry. For example, the viral latency protein EBNA-1 can lead to the formation of antibodies that are able to bind dsDNA [22]. High-titer antinuclear antibodies (ANA) are also associated with the presence of IgM or IgG against the EBV VCA [23] although our patient had undetectable ANA. Whether these antibodies carry the same immunologic and clinical significance as in systemic lupus erythematosus (SLE) is unknown, and this support the theory that dsDNA antibodies belong to a continuum where low avidity dsDNA antibodies can develop spontaneously and can be found in some healthy individuals while higher avidity antibodies are more specific for patients with clinical expression of SLE [24]. The occurrence of anti-dsDNA during anti-TNF therapy has been shown [25].

Treatment of EBV-associated LPDs depends on the patient's immune status and tumor pathology. Overall, in immunosuppressed patients and as exemplified in this case, withdrawal of the immunosuppressive insult is the mainstay for treatment; if this fails, rituximab is a potential addition to the treatment of B-cell LPDs in this setting [5]. The decision to add chemotherapy depends on the initial histologic diagnosis, with most high-grade B-cell lymphomas requiring combination regimens with addition of rituximab [5]. Furthermore, patients who fail an initial therapeutic trial with withdrawal of the drug and use of rituximab may require antiviral therapy in addition to chemotherapy, but this approach is still under investigation [5]. Recommendations for cases of iatrogenic EBV-associated LPDs after initiation of drugs are less well defined, but withdrawal of the trigger medication alone can reverse them. Hematopoietic stem cell transplantation in addition to immunochemotherapy is an effective approach for either CAEBV or HLH with malignant characteristics [26]. Those patients with concomitant HIV infection require the use of highly active antiretroviral treatment [5]. More specific therapies targeting EBV infected cells are under development, and these strategies include the use of gene therapy to enhance the sensitivity of EBV in latent phase to antiviral agents or to induce apoptosis of EBV infected cells, and infusion of autologous cytotoxic T lymphocytes specific against EBV latency proteins (CMD-003, baltaleucel-T) [27]. Another promising approach is the induction of the EBV lytic cycle, which increases sensitivity to antiviral medication, accomplished with the use of chemotherapy [27].

## 4. Conclusion

This case demonstrates that reactivation of EBV occurs in those patients with chronic immunologic derangements after initiation of immunomodulatory therapy. Even though parts of the molecular events related to EBV latency are known, the specific mechanisms through which anti-TNF agents are able to trigger the development of LPDs are not completely understood. Based on the current evidence, use of the EBV viral load in conjunction with flow cytometry analysis are helpful tools in detecting those patients with potentially curable diseases.
